# Characterization of volatile compounds of Pixian Douban fermented in closed system of gradient steady‐state temperature field

**DOI:** 10.1002/fsn3.2242

**Published:** 2021-03-29

**Authors:** Wenwu Ding, Yan Liu, Xiaoyan Zhao, Changbo Peng, Xiaoqing Ye, Zhenming Che, Yi Liu, Ping Liu, Hongbin Lin, Jiaquan Huang, Min Xu

**Affiliations:** ^1^ College of Food and Bioengineering Xihua University Chengdu China; ^2^ Sichuan Pixian Douban Company Limited Chengdu China

**Keywords:** aroma active compounds, gas chromatography‐olfactometry, gradient steady‐state temperature field, odor activity value, Pixian Douban

## Abstract

As an essential flavor condiment in Sichuan cuisine, Pixian Douban (PXDB) is usually produced by open fermentation process in strip pools or ceramic vats. In this study, an experiment of PXDB fermentation was conducted for 90 days in a closed system of gradient steady‐state temperature field (GSTF). To investigate the characterization of volatile compounds of PXDB in the closed system, the volatiles in three kinds of samples including samples of GSTF (SGT), samples of constant temperature (SCT), and samples of traditional fermentation (STF) were analyzed. The results showed that 75, 67, and 68 volatile compounds were detected in SGT, SCT, and STF, respectively. Compared with the traditional fermentation, the process in the closed system of GSTF was conducive to produce more kinds of esters and alcohols. A total of 22 major aroma active compounds were identified in three samples by combination analyses of gas chromatography‐olfactometry (GC‐O) and odor activity value (OAV). The appearance, smell, texture, and taste of the three different samples had shown different changes, but the sensory characteristics of the SGT were more similar to those of the STF by quantitative descriptive analysis (QDA) and principal component analysis (PCA). This study indicated that the closed system of GSTF could be applied in PXDB fermentation to obtain higher quality products, which brought a bright prospect of replacing the traditional fermentation process to realize the controllable industrialized production of PXDB.

## Graphical abstract

The results showed that flavor characteristics of SGT were similar to those of the STF. This study indicated that the closed system of GSTF could be applied in PXDB fermentation to obtain higher quality products, which brought a bright prospect of replacing the traditional fermentation process to realize the controllable industrialized production of PXDB.

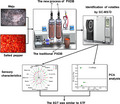

## 1 INTRODUCTION

As a traditional fermented food produced from a small town named Pixian County in Sichuan Province of China, Pixian Douban (PXDB) is an essential condiment in preparing Sichuan cuisine, which is honored as the spirit of Sichuan Cuisine and famous for its fascinating flavor globally. The production process of PXDB employed in most factories is traditional process, which has a history of hundreds of years. However, this process has many drawbacks, such as lower mechanization and automatization, lower productivity, higher labor and production cost, less quality and higher risks of food safety, which have seriously hindered the development of the industry (Ding et al., 2020). Therefore, with the development of fermentation technologies, it has become an urgent requirement to upgrade this industry by using advanced technologies and equipment.

**FIGURE 1:**
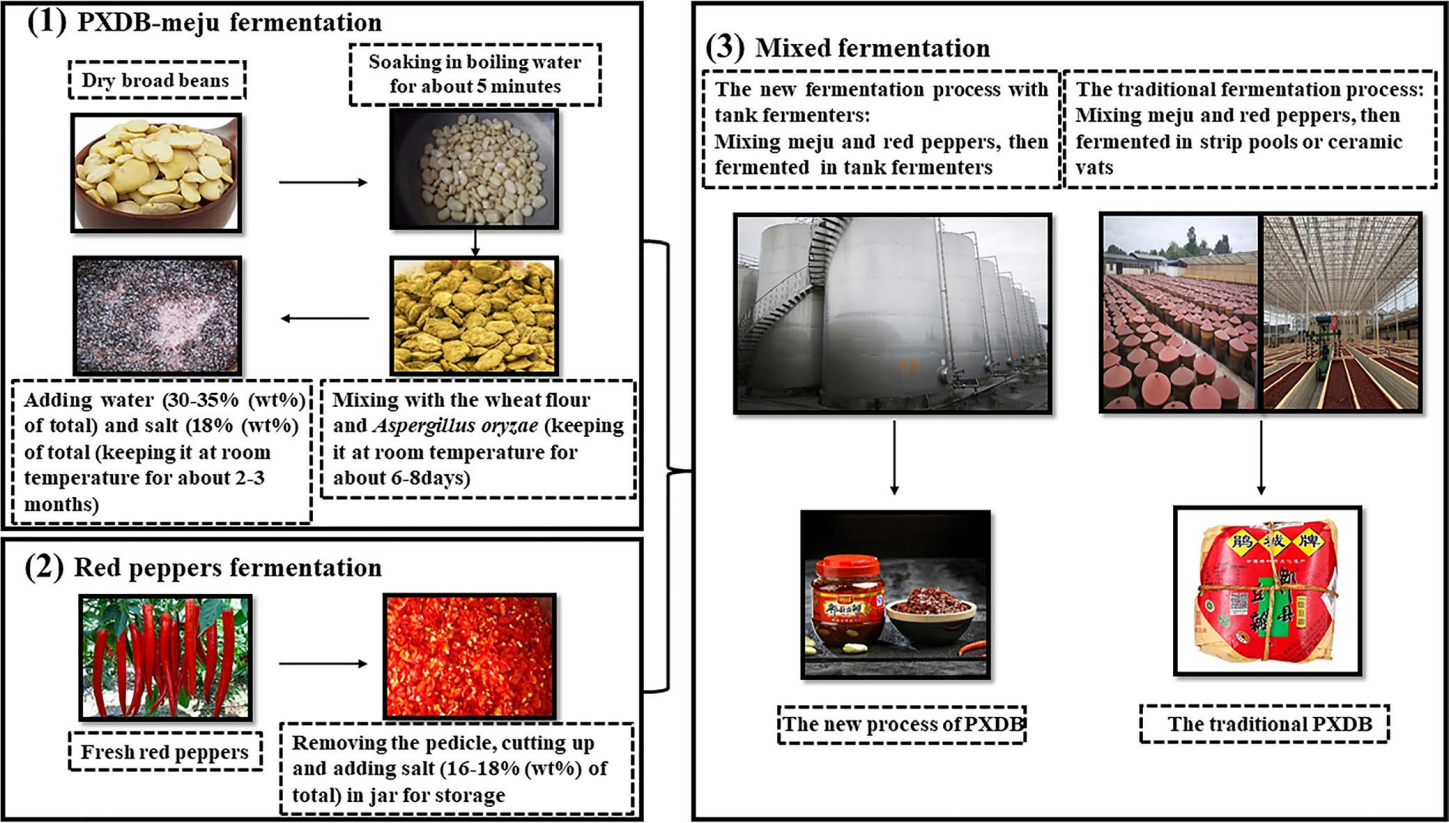
Process diagram of PXDB production

In recent two years, the production of PXDB in tank fermenter has been proposed and carried out in some factories. However, compared with traditional fermentation, this new process which just changed the equipment from strip pool or ceramic vat to tank fermenter without any other improvement is still dependent on the weather heavily (Figure 1). Moreover, the new process was not verified by the strict tests before it was amplified, which caused that the defects of the traditional process has not been overcome although the occupancy area of the factory had been greatly reduced by the new process (Ding et al., 2020). Therefore, as a new process with the most potential to replace the traditional fermentation, the PXDB process of tank fermenter should be further studied deeply to overcome the defects of traditional process and obtain high‐quality products before it was used in the factory.

Besides, aroma is a key indicator in evaluating the product quality of PXDB as the same other fermented foods such as soy sauces and vinegars (Al‐Dalali et al., 2019; Feng et al., 2014). Therefore, the new process should be developed based on the study of traditional fermentation process to obtain high‐quality product with good aroma. In the literatures, many studies about the volatile compositions of PXDB have been conducted, and many valuable results were obtained (Li et al., 2016, 2018, 2019; Lin et al., 2019). In Lu's study, 22 key volatile compounds were identified in PXDB fermented with traditional process (Lu et al., 2019). The microbial communities, flavors, and their relationships in PXDB were studied in Liu's study, which revealed that the flavor compounds would accumulate significantly with the prolonging of the fermentation period, especially for umami‐taste amino acids, organic acids, and volatile compounds (Liu et al., 2020). In Lin's study, 21 aroma active compounds were detected with flavor dilution factor ranging from 2 to 16 in PXDB (Lin et al., 2019). Those researches were benefit of revealing the aroma of the traditional fermentation products and provided the foundation for the studies of new process.

In this study, a closed system of PXDB fermentation under gradient steady‐state temperature field (GSTF) was constructed based on the results of our previous research (Ding et al., 2020). Then, three experiments of PXDB fermentation were conducted in the closed system of GSTF, closed system of constant temperature, and traditional fermentation system, respectively. The volatiles of the product fermented in the new system were characterized by comparing with those of the products from the closed system of constant temperature and the traditional fermentation system in order to clarify the volatile compounds of the three products and improve the new process to produce high‐quality products.

## 2 MATERIALS AND METHODS

### 2.1 Procedures of three different fermentation processes

**FIGURE 2:**
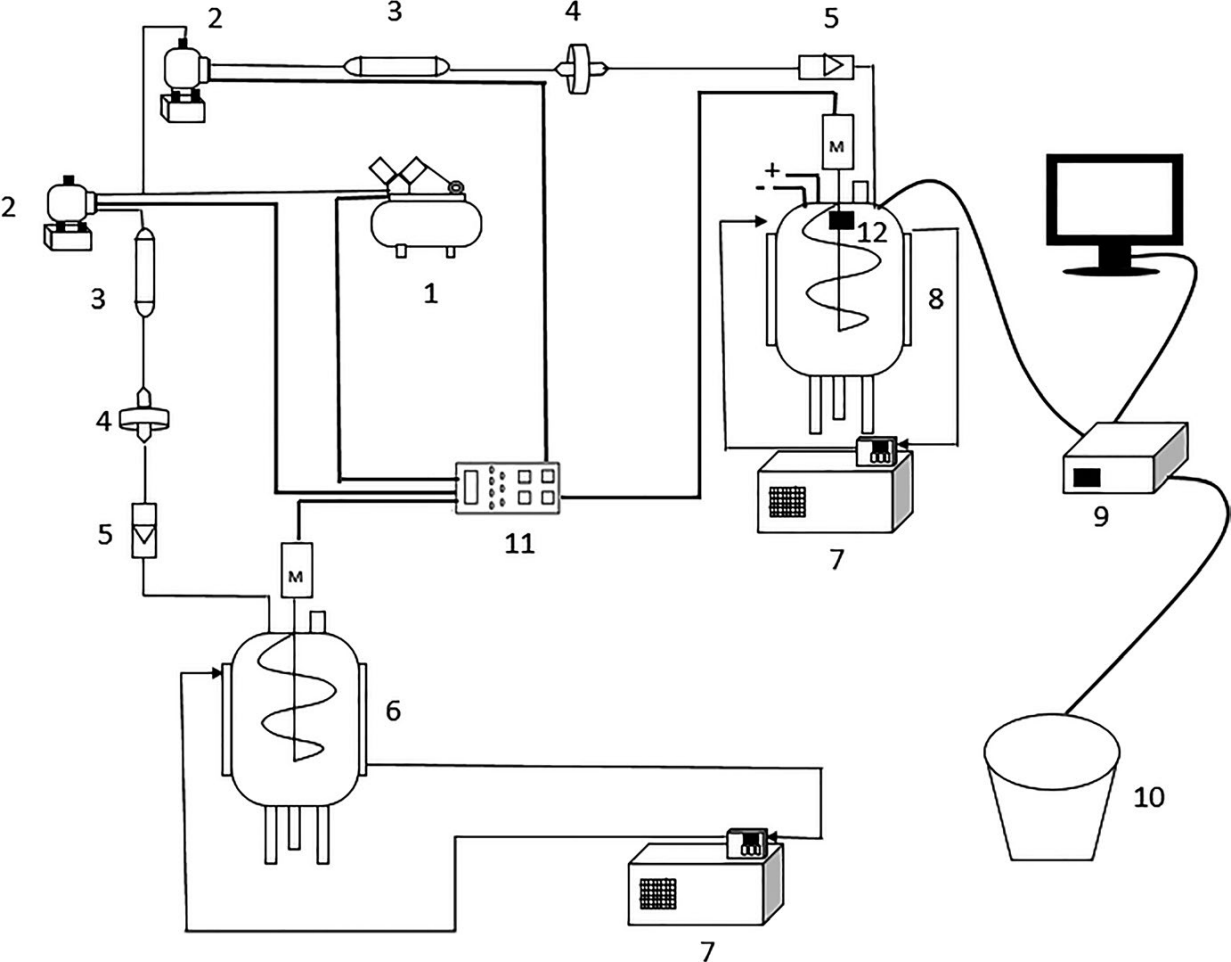
Schematic apparatus of three fermentation systems for PXDB. (1) pump; (2) solenoid valve; (3) air dryer; (4) air filter; (5) gas rotameter; (6) constant temperature fermenter; (7) temperature control unit; (8) GSTF fermenter; (9) temperature detected unit; (10) ceramic vat; (11) timing socket; (12) intelligent temperature controller

The schematic apparatus of fermentation processes of PXDB is shown in Figure 2. As shown in Figure 2, the apparatus of the new closed system was mainly constructed from two tank fermenters of 50 L, an air supply system, and a thermostat. The fermentation of PXDB in closed system was conducted with the fermenters of a filling coefficient of 0.8 at constant temperature of 30°C and at GSTF, respectively. It should be noted that the GSTF in the fermenter was designed and achieved by keeping the temperature of jacket at 27°C and the propeller temperature between 45°C and 52°C. The traditional fermentation was carried out in an open ceramic vat of 30 L following the traditional operation. The meju and red peppers purchased from Sichuan PXDB Co., LTD. were first mixed at a ratio of 1:3 before starting the fermentation and then transfer into the fermenters and the ceramic vats. The PXDB in the tank fermenters was stirred once every 6 hr at a rate of 35 rpm for 3 min and ventilated every 4 hr at a rate of 5 L/min for 5 min. The experiment was carried out for 90 days from August to October.

### 2.2 Extraction of volatile compounds

The HS‐SPME sampling was carried out according to previously described method with some modifications (Lu et al., 2019). Sample was mashed into homogenized paste, and 5.0‐g mashed sample was put into a 15‐ml solid‐phase micro‐extraction (SPME) vial, where 10 μl of 1,2‐dichlorobenzene (10 μg/ml in methanol) was used as the internal standard. Then, the vial was sealed and equilibrated at 55°C for 30 min. Afterwards, a carboxen‐polydimethylsiloxane fused silica (CAR/PDMS, 75 μm)‐coating fiber (Anpel Inc.) was exposed to the headspace of the sample to absorb the volatiles at 55°C for 40 min and the coating fiber was quickly inserted into a GC injection port and desorbed at 250°C for 5 min.

### 2.3 Gas chromatography‐mass spectrometer analysis

GC‐MS analysis was performed on a Shimadzu gas chromatograph (Shimadzu). Separation of volatile components was performed on a DB‐5MS column (Agilent). Helium was used as a carrier gas at a constant flow rate of 1 ml/min. Oven temperature was maintained at 40°C for 1 min, programmed at 5°C/min to 100°C and held for 1 min, programmed at 7°C/min to 150°C and held for 4 min, thereafter programmed at 5°C/min to 185°C and held for 5 min, finally programmed at 10°C/min to 200°C. The mass spectrometer was operated in electron impact mode with the electron energy set at 70 eV and a scan range of 35–500 m/z. The temperature of MS source was set at 200°C. A mixture of n‐alkanes (C6–C20) was injected directly into GC‐MS under the same condition as that for the samples to calculate the retention indices (RIs). Each volatile compound was identified using the National Institute of Standards and Technology (NIST17) library. The content of each compound is obtained by comparing it with the internal standard.

### 2.4 Gas chromatography‐olfactometry analysis

The samples were analyzed by using a gas chromatograph equipped with an olfactory detector (Shimadzu). The sample was injected in the splitless mode at 200°C for 5 min. The oven temperature was adjusted to the same condition as that of GC‐MS. Retention times and descriptions of aromas were recorded by three trained assessors (replaced at 10 min intervals) after the sample injection. Each trained assessor sniffed each sample in three replicates.

### 2.5 Qualification and quantification of volatile compounds

The qualification of the volatile compounds was done of the following methods. The compounds were tentatively identified by comparing their mass spectra with those which were found in the National Institute of Standards and Technology library (NIST17), and by comparing their RIs with those reported in the previous literature. Quantitative analysis of volatile compounds was conducted under the same condition as that of GC‐MS. The chemical quantities were calculated based on the relative peak area to the area of 1,2‐dichlorobenzene, which was used as the internal standard.

### 2.6 Calculation of odor activity values

Although GC‐O analysis is an effective method for odorant identification, it could not indicate a final importance of the odorant to the overall aroma (Gao et al., 2014). Therefore, the ultimate contribution of a particular compound to the overall aroma of PXDB mainly determined its odor threshold and the odor activity value (OAV) (Giri et al., 2010). If the OAV of the compound calculated as the ratio of its concentration to odor threshold was larger than or equal to 1, it could be considered to contribute to the overall aroma (Zhao et al., 2020).

### 2.7 Sensory evaluation

#### 2.7.1 Establishment of flavor descriptors

The sensory characteristics of the three kinds of samples were evaluated by the quantitative descriptive analysis (QDA). According to the methods previously described (Chen et al., 2013; Gao et al., 2018; Zhao et al., 2020), the sensory characteristics of samples were characterized by the sensory team, which was composed of 10 students (five males and five females, aged 23–26) with rich sensory evaluation experience from Xihua University. Firstly, the evaluators discussed the aroma characteristics and put forward the descriptors of PXDB. Then, a flavor description table was obtained according to the descriptors proposed by the evaluators and in the literature. Finally, the evaluator tasted three kinds of samples and retained the flavor descriptors with a pass rate of more than 50% to establish the final sensory description vocabulary.

#### 2.7.2 Evaluation method

10 g PXDB was taken into a 30‐ml plastic cup and coded randomly. The intensity range of aromas were 0–9 scale (0 represented none, 9 represented very strong). The sensory evaluation of each sample was tested three times and the results were averaged.

### 2.8 Statistical analysis

All data were presented as means ± *SD* for at least three replicates. The graph presentations were generated using Origin version 8.5 (OriginLab Inc.). Principal component analysis (PCA) was performed using the Simca version 14.1 (Simca Inc.).

## 3 RESULTS AND DISCUSSION

### 3.1 Identification and quantification of volatile compounds by GC‐MS

**FIGURE 3:**
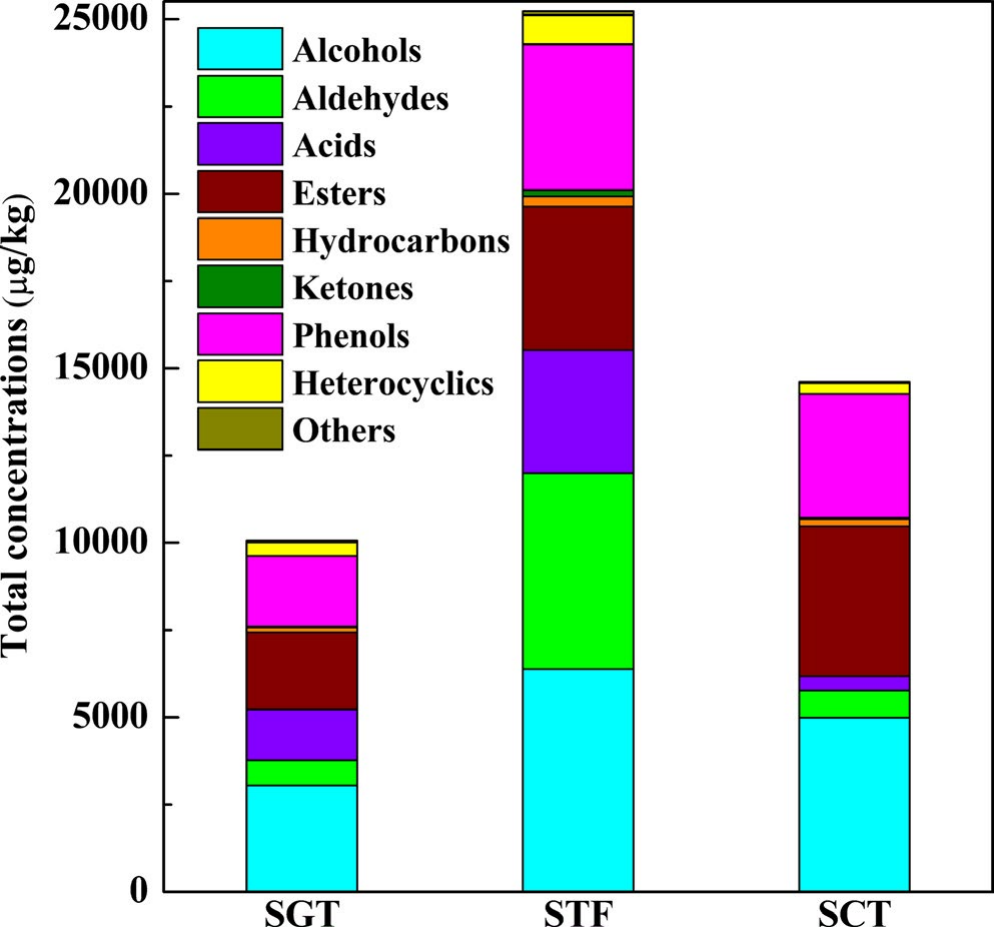
Total concentrations of volatile compounds in three samples

The volatile compounds in the samples of gradient temperature of steady‐state (SGT), samples of constant temperature (SCT), and samples of traditional fermentation (STF) were identified by GC‐MS. As a result, a total of 103 volatiles were identified in the three samples (Table 1), which could be categorized into 10 different groups, including alcohols, aldehydes, acids, esters, hydrocarbons, ketones, phenols, heterocyclics, and others. As shown in Figure 3, alcohols, acids, esters, and phenols were the largest four groups in SGT accounting for approximately 6.1%, 2.9%, 4.4%, and 4.0% of the total volatiles, respectively. For SCT, alcohols, esters, and phenols were the three largest groups accounting for approximately 10.0%, 8.6%, and 7.1% of the total volatiles, respectively. For STF, alcohols, aldehydes, acids, esters, and phenols were the dominant classes, and accounted for 12.8%, 11.3%, 8.2%, and 8.4% of the total volatiles, respectively.

**TABLE 1:** Identification and quantification of volatile compounds in three samples

No.	Compounds	CAS	Concentration (µg/kg)^a^			Previously reported
			SGT	STF	SCT	
Alcohols						
1	Phenethyl alcohol	60–12–8	1,162.2 ± 1.04	1564.3 ± 0.12	2,808.3 ± 1.44	1,2,5–7
2	Furfuryl alcohol	98–00–0	977.7 ± 0.06	1821.8 ± 0.11	1,294.0 ± 0.80	1,7
3	Linalool	78–70–6	125.7 ± 0.03	375.7 ± 0.04	191.5 ± 0.1	1,6
4	Benzyl alcohol	100–51–6	146.2 ± 0.08	214.8 ± 0.03	165.8 ± 0.09	1,6,7
5	1‐Hexanol	111–27–3	149.8 ± 0.04	720.0 ± 0.05	186.6 ± 0.11	4,7
6	2,3‐Butanediol	513–85–9	53.1 ± 0.06	635.2 ± 0.08	7.8 ± 0.06	3
7	(‐)‐Alpha‐terpineol	10482–56–1	nd	223.2 ± 0.004	nd	1
8	3‐Methylthiopropanol	505–10–2	186.7 ± 0.07	458.5 ± 0.16	287.2 ± 0.13	3
9	2‐Heptanol	543–49–7	27.0 ± 0.07	81.3 ± 0.013	28.8 ± 0.06	b
10	1‐Octen−3‐ol	3391–86–4	nd	165.4 ± 0.05	nd	b
11	1‐Heptanol	111–70–6	nd	80.9 ± 0.07	nd	b
12	Trans−2‐octen−1‐ol	18409–17–1	nd	32.9 ± 0.06	nd	b
13	2‐Phenyl−2‐propanol	617–94–7	139.4 ± 0.05	nd	nd	b
14	3‐Methyl−1‐pentanol	589–35–5	24.6 ± 0.04	nd	nd	b
15	2‐(4‐Methylphenyl) propan−2‐ol	1197–01–9	14.9 ± 0.03	nd	nd	b
16	(5‐Methyl−2‐furyl) methanol	3857–25–8	13.2 ± 0.06	nd	nd	b
17	Beta‐ethylphenethyl alcohol 98	2035–94–1	12.0 ± 0.05	nd	nd	b
18	(‐)‐Verbenone	1196–01–6	10.7 ± 0.04	nd	nd	b
19	Diisobutylcarbinol	108–82–7	nd	nd	18.1 ± 0.06	b
Aldehydes						
20	Phenylacetaldehyde	122–78–1	459.0 ± 0.03	1815.4 ± 0.06	482.8 ± 0.05	1–3,5–7
21	Benzaldehyde	100–52–7	83.7 ± 0.04	1979.5 ± 0.9	94.1 ± 0.06	1,3,6,7
22	Furfural	98–01–1	34.6 ± 0.08	411.6 ± 0.03	34.4 ± 0.03	1,3,6
23	3‐(Methylthio)propionaldehyde	3268–49–3	90.7 ± 0.09	369.4 ± 0.05	77.5 ± 0.07	8
24	1‐Nonanal	124–19–6	36.6 ± 0.06	252.6 ± 0.03	68.1 ± 0.08	3,5,6
25	3‐Methyl−2‐butenal	107–86–8	nd	185.0 ± 0.07	23 ± 0.04	b
26	Octanal	124–13–0	nd	44.7 ± 0.05	nd	b
27	Decanal	112–31–2	nd	39.6 ± 0.06	nd	5
28	Trans−2‐heptenal	18829–55–5	nd	149.9 ± 0.07	nd	b
29	Heptaldehyde	111–71–7	nd	137.9 ± 0.11	nd	b
30	2‐Phenyl−2‐butenal	4411–89–6	nd	127.5 ± 0.05	nd	10
31	(e)−2‐Octenal	2548–87–0	nd	64.9 ± 0.04	nd	b
32	Cocal	21834–92–4	nd	36.6 ± 0.06	nd	b
33	Trans−2‐methyl−2‐butenal	497–03–0	17.9 ± 0.004	nd	nd	b
Acids						
34	Isovaleric acid	503–74–2	567.0 ± 0.04	1,002.9 ± 0.04	nd	1
35	Butyric acid	107–92–6	311.3 ± 0.03	954.6 ± 0.05	nd	2,7
36	Hexanoic acid	142–62–1	192.1 ± 0.02	681.8 ± 0.06	133 ± 0.03	2,7
37	4‐Methylvaleric acid	646–07–1	27.8 ± 0.04	nd	nd	2,7
38	Octanoic acid	124–07–2	15.4 ± 0.06	nd	nd	2
39	2‐Methyl butyric acid	116–53–0	214.8 ± 0.07	517 ± 0.03	201.7 ± 0.04	11
40	Isobutyric acid	79–31–2	125.7 ± 0.12	371.5 ± 0.08	61.9 ± 0.06	1
Esters						
41	Ethyl hexanoate	123–66–0	165.8 ± 0.04	210.0 ± 0.06	373.1 ± 0.06	2,4,5,7
42	Ethyl butyrate	105–54–4	168.8 ± 0.06	672.0 ± 0.04	325.5 ± 0.05	2,7
43	Ethyl laurate	106–33–2	265.4 ± 0.08	161.0 ± 0.06	662.3 ± 0.07	5
44	Methyl palmitate	112–39–0	170.9 ± 0.05	383 ± 0.05	178.2 ± 0.02	2,5
45	Ethyl myristate	124–06–1	182.5 ± 0.04	142.3 ± 0.11	415.9 ± 0.08	2,5–7
46	Methyl salicylate	119–36–8	102.8 ± 0.05	305.0 ± 0.08	172.7 ± 0.03	6
47	Ethyl isovalerate	108–64–5	143.8 ± 0.04	294.9 ± 0,003	205.7 ± 0.05	7
48	Ethyl phenylacetate	101–97–3	117.0 ± 0.08	264.9 ± 0.1	319.0 ± 0.13	1,3–7
49	Methyl laurate	111–82–0	117.7 ± 0.07	209.1 ± 0.06	158.7 ± 0.05	5
50	Ethyl 2‐methylbutyrate	7452–79–1	75.6 ± 0.07	135.2 ± 0.09	155.3 ± 0.05	2,7
51	Isoamyl acetate	123–92–2	56.6 ± 0.08	144.2 ± 0.03	61.8 ± 0.04	7
52	Ethyl trans−4‐decenoate	76649–16–6	42.8 ± 0.05	58.0 ± 0.03	153.9 ± 0.04	5
53	Ethyl linoleate	544–35–4	nd	168.5 ± 0.05	nd	2,3,5,6
54	Ethyl valerate	539–82–2	nd	nd	58.8 ± 0.05	2,7
55	Ethyl nonanoate	123–29–5	17.3 ± 0.05	nd	42.6 ± 0.06	7
56	Ethyl heptanoate	106–30–9	13.0 ± 0.03	nd	38.4 ± 0.08	7
57	N‐pentadecanoic acid ethyl ester	41114–00–5	12.1 ± 0.02	nd	33.7 ± 0.04	7
58	Ethyl caprylate	106–32–1	nd	nd	221.7 ± 0.02	2,5,7
59	Diethyl succinate	123–25–1	nd	nd	23.0 ± 0.03	2,7
60	Methyl nonanoate	1731–84–6	nd	nd	9.8 ± 0.02	b
61	Ethyl methacrylate	7493–69–8	81.2 ± 0.05	nd	nd	b
62	Methyl phenylacetate	101–41–7	17.9 ± 0.02	nd	nd	b
63	Gamma‐butyrolactone(gbl)	96–48–0	7.5 ± 0.04	nd	nd	b
64	Allyl 2‐ethylbutyrate	7493–69–8	8.0 ± 0.05	100.9 ± 0.02	nd	b
65	Ethyl L (‐)‐lactate	687–47–8	45.0 ± 0.05	201.2 ± 0.05	74.7 ± 0.04	b
66	Methyl myristate	124–10–7	93.2 ± 0.12	165.1 ± 0.08	99.5 ± 0.06	b
67	Caprylic acid methyl ester	111–11–5	50.2 ± 0.07	120.3 ± 0.05	37.5 ± 0.04	b
68	Methyl hexanoate	106–70–7	28.4 ± 0.05	111.2 ± 0.05	30.9 ± 0.03	b
69	Ethyl isobutyrate	97–62–1	31.4 ± 0.05	45.6 ± 0.04	59.8 ± 0.05	b
70	Dihydro−5‐methyl−5‐vinylfuran−2(3h)‐one	1073–11–6	25.4 ± 0.04	60.2 ± 0.05	24.2 ± 0.02	b
71	Ethyl crotonate	623–70–1	16.3 ± 0.03	68.4 ± 0.05	38.4 ± 0.02	b
72	Methyl decanoate	110–42–9	13.5 ± 0.04	47.9 ± 0.05	30.7 ± 0.05	b
73	2‐Methylbutyl acetate	624–41–9	17.7 ± 0.13	43.3 ± 0.11	24.8 ± 0.04	b
74	Ethyl caprate	110–38–3	46.3 ± 0.12	nd	66.5 ± 0.02	5,11,12
75	Phenethyl acetate	103–45–7	20.8 ± 0.05	nd	36.9 ± 0.12	b
76	Gamma‐nonanolactone	104–61–0	19.0 ± 0.09	nd	17.5 ± 0.005	11
77	Ethyl undecanoate	627–90–7	13.8 ± 0.03	nd	124.1 ± 0.04	b
78	Palmitoleic acid methyl ester	1120–25–8	15.7 ± 0.11	nd	25.8 ± 0.01	b
Hydrocarbons						
79	Tetradecane	629–59–4	66.7 ± 0.06	nd	47.3 ± 0.05	2
80	N‐hexadecane	544–76–3	61.9 ± 0.04	39.9 ± 0.03	56.3 ± 0.04	b
81	Octadecane	593–45–3	nd	127.0 ± 0.05	nd	b
82	1‐Chloropentane	543–59–9	nd	19.1 ± 0.04	nd	b
83	N‐hexacosane	630–01–3	nd	15.1 ± 0.06	nd	b
84	1‐Chlorooctadecane	3386–33–2	nd	88.3 ± 0.08	42.2 ± 0.06	b
85	1‐Chloro−3‐methylbutane	107–84–6	nd	nd	37.8 ± 0.06	b
86	1‐Iodododecane	4292–19–7	nd	nd	23.3 ± 0.05	b
87	N‐heptadecane	629–78–7	15.2 ± 0.13	nd	nd	b
Ketones						
88	2‐Heptanone	110–43–0	17.8 ± 0.11	74.9 ± 0.12	23.7 ± 0.04	7
89	2‐Nonanone	821–55–6	nd	26.4 ± 0.05	16.4 ± 0.08	7
90	2,6,6‐Trimethyl−2‐cyclohexene−1,4‐dione	1125–21–9	nd	53.4 ± 0.11	nd	b
91	Isophorone	78–59–1	9.5 ± 0.08	25.6 ± 0.07	nd	b
Phenols						
92	4‐Ethyl−2‐methoxyphenol	2785–89–9	1,082.9 ± 0.2	2,299.6 ± 0.38	2020.6 ± 0.3	1,2,5–7
93	4‐Ethylphenol	123–07–9	823.7 ± 0.13	1759.6 ± 0.15	1,402.4 ± 0.11	1,2,5–7
94	Guaiacol	90–05–1	63.4 ± 0.06	121.1 ± 0.05	76.4 ± 0.04	8
95	P‐cresol	106–44–5	45.3 ± 0.04	nd	55.3 ± 0.05	4
Heterocyclics						
96	2‐Acetyl pyrrole	1072–83–9	350.4 ± 0.02	837.7 ± 0.12	278.2 ± 0.04	7
97	2‐Acetylfuran	1192–62–7	15.2 ± 0.05	nd	16.8 ± 0.05	5,7
98	2,6‐Dimethylpyrazine	108–50–9	15.2 ± 0.04	nd	6.7 ± 0.03	1–3,5,7
99	2‐Propionylfuran	3194–15–8	13.4 ± 0.05	nd	nd	b
Others						
100	Toluene	108–88–3	38.9 ± 0.04	29.3 ± 0.05	8.5 ± 0.02	2
101	M‐xylene	108–38–3	nd	79.8 ± 0.005	nd	2
102	Borneol	507–70–0	nd	nd	28.1 ± 0.07	b
103	Tetramethylthiourea	2782–91–4	22.2 ± 0.06	nd	nd	b

Note

Values expressed as average (*n* = 3) ± standard deviation.

Abbreviation: GSTF, gradient steady‐state temperature field; nd, not detected; SCT, samples of constant temperature; STF, samples of traditional fermentation; SGT, samples including samples of GSTF.

^a^ Relative peak area to that of internal standard (10 μl of 10 μg/ml 1,2‐dichlorobenzene in methanol) using DB‐5MS column.

^b^ Compounds by identified in fermented soybean products.

^1^ Zhang et al. (2020).

^2^ Jo, et al. (2011).

^3^ Zhao et al. (2011).

^4^ Inoue et al. (2016).

^5^ Li et al. (2018).

^6^ Li et al. (2016).

^7^ Lee and Ahn (2009).

^8^ Lu et al. (2019).

^9^ Giri et al. (2010).

^10^ Al‐Dalali et al. (2019).

^11^ Lu et al. (2020).

**FIGURE 4:**
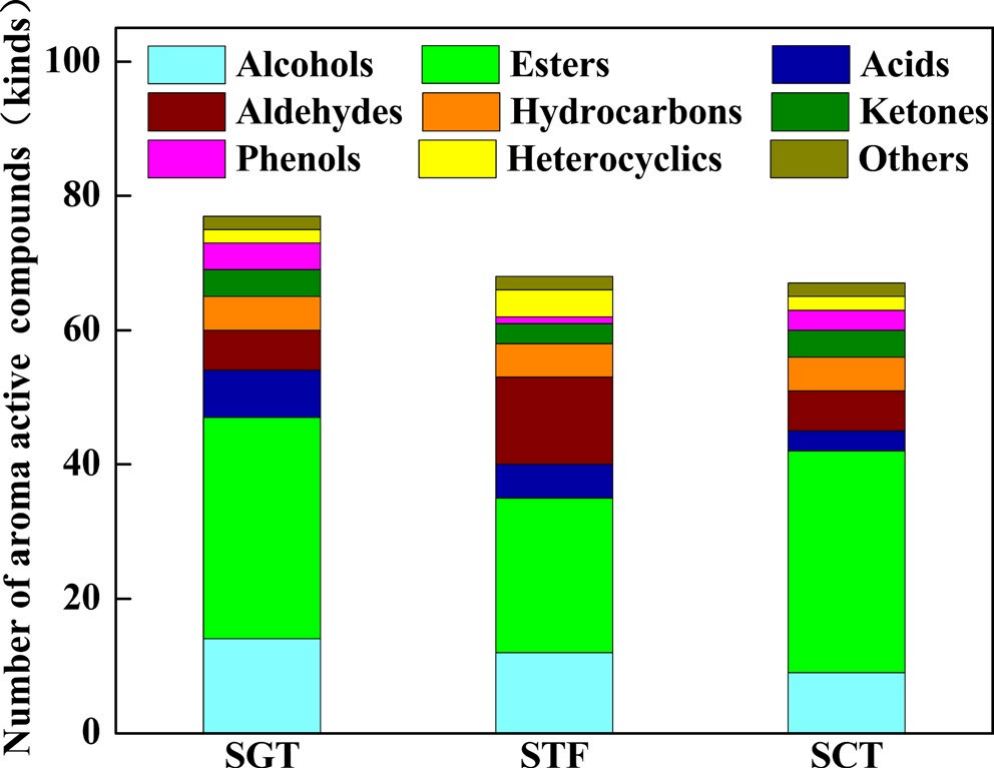
Number of volatile compounds in three samples

As shown in Figure 4, there were 75, 67, and 68 kinds of volatile compounds in SGT, SCT, and STF, respectively. Compared with traditional fermentation, the number of esters in SGT and SCT was larger while the number of aldehydes was smaller, which implied that SGT and SCT both had an advantage of producing esters rather than aldehydes. Besides, more kinds of esters and acids were included in the SGT but there were more kinds of aldehydes in the STF. The reasons for this phenomenon might be that the traditional fermentation process could provide more various fermentation conditions, which were conducive to the oxidation of alcohols to aldehydes, while the closed system of tank fermenter with stable temperature could provide more energy for some biochemical reactions among small molecules, which would promote more alcohols to participate in the esterification reaction of producing more esters. Notably, alcohols were easily formed in the SGT and STF probably because the complexion of temperature in the GSTF was more similar with that in the traditional fermentation. Meanwhile, alcohols have played important roles in the flavor characteristics of PXDB (Li et al., ,^2016^, ^2018^; Lu et al., 2020).

Esters are highly important indicators for the quality grade of fermentation products (Moy et al., 2012), which might mainly be formed by alcohol fermentation or esterification between acids and alcohols during the aging technology (Charles et al., 2000; Li et al., 2018). In this work, ester was also the largest group of all the volatiles as reported in other studies of fermented foods, in which 38 volatile esters were identified in a concentration ranging from 7.5 to 672 µg/kg (Table 1). Totally, 33, 33, and 23 volatile esters were contained in SGT, SCT, and STF, respectively, indicating that the fermentation processes of closed system were beneficial to the formation of ester species. Nineteen of the 38 identified volatile esters in this study were also found previously in other fermented soybean products as marked in Table 1. Among the identified 38 volatile esters, 21 esters were shared in those three samples and the other 17 esters were identified in one or two samples suggesting that these compounds were unique for PXDB. For example, ethyl valerate, ethyl linoleate, and ethyl methacrylate only existed in SCT, STF, and SGT, respectively, but nonanoate in SCT and SGT and allyl 2‐ethylbutyrate in STF and SGT. Besides, the top four esters in these three samples are different showing that those in SCT were ethy laurate, ethy myristate, ethy hexanoate, and ethy butyrate but ethy butyrate, methyl palmitate, methyl salicylate, ethy isovalerate, and ethy phenylacetate in STF and ethy laurate, ethy myristate, ethy butyrate, and ethy hexanoate in STG. In addition, it has been reported that many lactones have an aroma of pleasant and a low threshold (Zhou et al., 2017). Therefore, gamma‐nonanolactone which was detected in SGT (19.0 µg/kg) and SCT (17.5 µg/kg) could make a positive contribution to the overall aroma of the samples although its concentration was not high.

A total of 19 volatile alcohols that might mainly come from alcohol fermentation were identified becoming the second largest group of the volatile compounds, in which seven of them had been reported in the literatures. Besides, eight alcohols were shared in those three samples, in which concentrations were the lowest in SGT but highest in STF. The three samples showed different quantities and concentrations of volatile alcohols. In total, 14, 8 and 12 volatile alcohols were found in SGT, SCT, and STF with their concentrations ranging from 10.7 to 1,162.2 µg/kg, 7.8 to 2,808.3 µg/kg, and 32.9 to 1,821.8 µg/kg, respectively. Among the alcohols, phenethyl alcohol showed the highest concentration in SGT and SCT, but furfuryl alcohol in STF. Particularly, fusel alcohols, such as 2‐phenyl‐1‐propanol, 3‐methyl‐1‐pentanol, and (5‐methyl‐2‐furyl) methanol, were identified in appreciable abundance in SGT in this study, which were not appeared in other two samples. The changes of alcohol compounds might be attributed to the complicated metabolism of some microorganisms. For instance, *Lactobacillus plantarum* was potential to hydrolyze proteins and generate phenylethyl alcohol (Wang et al., 2017).

Fourteen volatile aldehydes were obtained in the three samples becoming the third largest group of the identified volatiles, which might be resulted from alcohol oxidation (Chinnici et al., 2009). Among the identified aldehydes, 13 were found in STF much larger than 6 in SGT and SCT. Five of the 14 volatile aldehydes identified in this work were reported in the literatures including phenylacetaldehyde, benzaldehyde, furfural, 1‐nonanal, and decanal. Besides, as shown in Table 1, the volatiles of benzaldehyde, phenylacetaldehyde, and 3‐(methylthio) propionaldehyde were the most three abundant aldehydes in SGT and SCT, while benzaldehyde, phenylacetaldehyde, and furfural were the most three abundant aldehydes in STF. The phenylacetaldehyde was generally considered to be produced by a series of biochemical reactions such as the oxidation of phenethyl alcohol (Wang et al., 2017), of which concentration was determined at 459, 482.8, and 1,815.4 µg/kg in SGT, SCT, and STF, respectively. From above analysis, it could be knew that the quantities and concentrations of volatile aldehydes in STF were both higher than those in the other two samples.

Seven, three, and five acid volatiles were identified in SGT, SCT, and STF, respectively, ranked as the fourth largest group, in which five of them had been reported previously. The concentrations of the acids in SGT, SCT, and STF were in a range from 15.4 to 567 µg/kg, 61.9 to 132.6 µg/kg, and 371.5 to 1,002.9 µg/kg, respectively. The volatile acid of the highest concentration was isovaleric acid both in SGT and STF but 2‐methyl butyric acid in SCT. It was worth mentioning that isovaleric acid and butyric acid both appeared in SGT and STF while 4‐methylvaleric acid and octanoic acid were only found in SGT, and the other acids were all detected in the three samples. This phenomenon indicated that, in terms of species, SGT is more similar to STF than SCT.

Four phenols were detected in all the samples, of which the phenol compounds of the highest and lowest concentration were 4‐ethyl‐2‐methoxyphenol and p‐cresol, respectively. Three phenols were detected in STF, which concentrations were ranged from 121.1 µg/kg to 2,299.6 µg/kg. P‐cresol was only found both in SGT and SCT, but the other three phenols were identified in all the three samples. According to the reports, the phenolic compounds might mainly come from the lignin degradation (Lu et al., 2019; NATERA et al., 2003). Besides, guaiacol and 4‐ethylphenol also existed naturally in the PXDB (Lu et al., 2019). 4, 3, and 1 heterocyclic compounds were identified in SGT, SCT, and STF, respectively, of which the concentration of 2‐acetyl pyrrole was the highest in the three samples. Furthermore, the concentration of 2,6‐dimethylpyrazine in SGT was twice as that in SCT. 2‐acetylfuran and 2,6‐dimethylpyrazine were found both in SGT and SCT, while 2‐propionylfuran only appeared in SGT.

For hydrocarbons, five compounds were obtained in SCT and STF but three in SGT. n‐hexadecane was found in all the three samples, while the other hydrocarbons were detected only in one or two samples. Table 1 showed that the concentrations of five hydrocarbons were ranged from 15.2 to 66.7 µg/kg, 23.3 to 56.3 µg/kg, and 15.1 to 127 µg/kg in SGT, SCT, and STF, respectively. For ketones, two compounds were obtained in SGT and SCT but four in STF, of which the concentration of 2‐heptanone was the highest in the three samples.

Therefore, compared with the traditional fermentation, more kinds of esters and alcohols were easily formed in the SGT, which might account for the aroma differences between these two samples. To characterize the aroma of the SGT, it was necessary to screen the aroma active compounds of the three kinds of samples by GC‐O.

### 3.2 Characterization of the aroma active compounds by GC‐O

**FIGURE 5:**
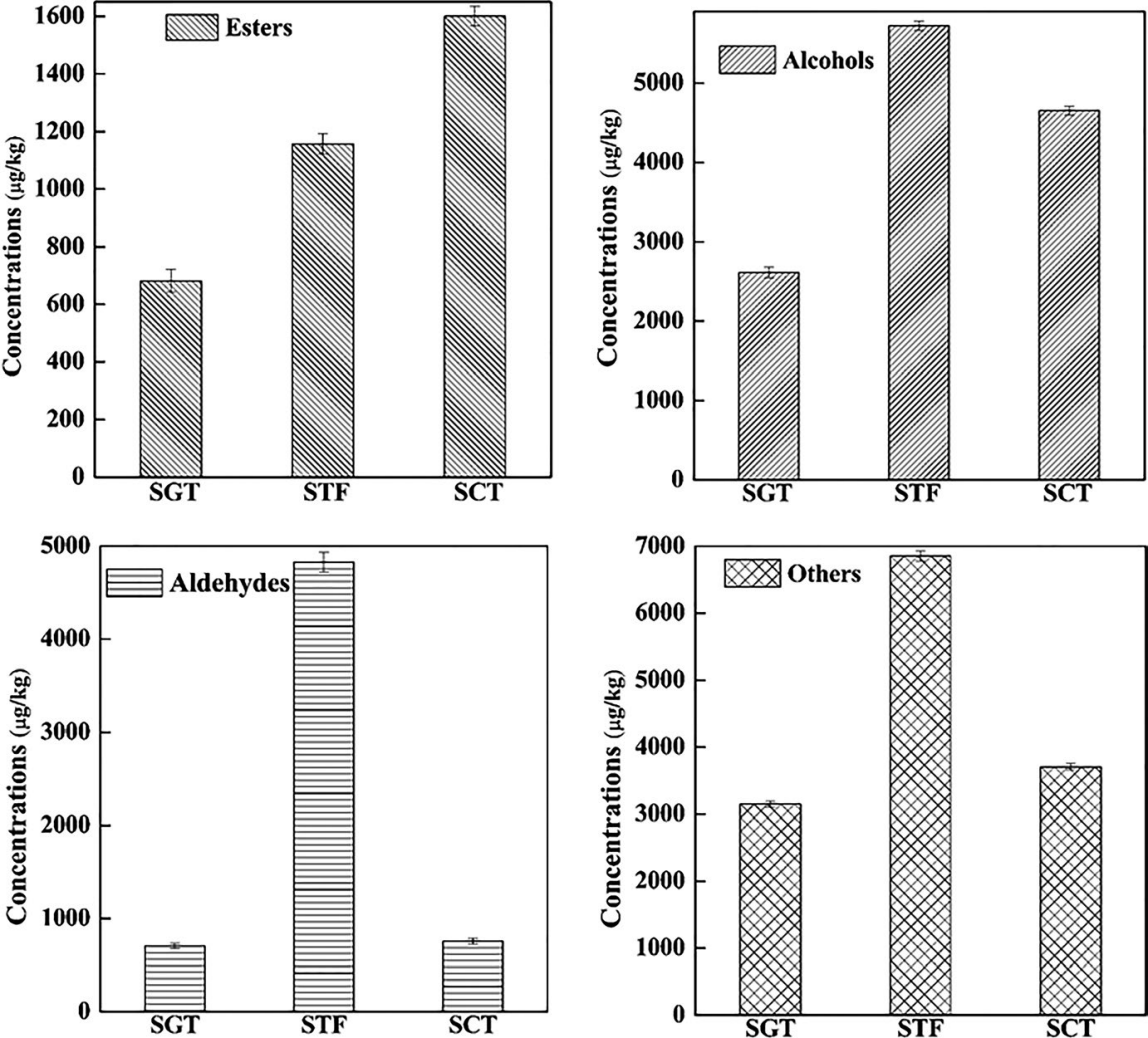
Concentrations of each class of aroma‐active compounds in three samples

**FIGURE 6:**
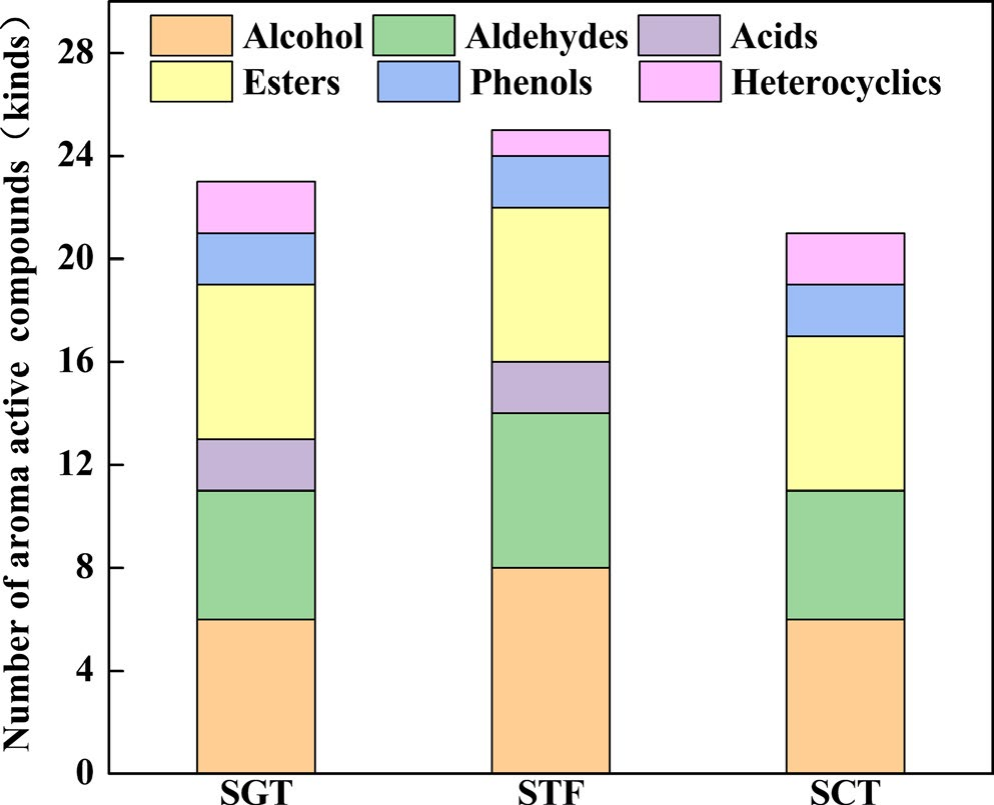
Number of aroma active compounds in three samples

In order to further characterize the important volatile components in the three kinds of samples, the aroma extracts obtained by HS‐SPME were subjected to GC‐O analysis. As shown in Figures 5 and 6, alcohols, esters, and aldehydes were the three largest major volatile compounds in the three samples, which were consistent with the results of the previous study (Wu et al., 2018). As shown in Table 2, a total of 27 active compounds detected by HS‐SPME/GC‐O could be grouped into sweet, burnt, sour, honey, bread, fruity, floral, green leaves, almonds, pickles, baked potatoes, sweat, mint, and nuts, which were associated with different chemical groups such as alcohols, aldehydes, acids, esters, phenols, pyrroles, and pyrazines. Among the active aromas, the compound of the highest concentration was phenethyl alcohol (honey‐like) in the SGT and SCT but 4‐Ethyl‐2‐methoxyphenol (burnt, spicy) in the STF. Besides, there were 20 aroma active volatiles that were shared in the three samples, while the other volatiles were found only in one or two samples. For example, the aroma‐active compounds including alpha‐terpineol (oil, anise, and mint), 1‐octen‐3‐ol (earthy), decanal (floral, sweet), and ethyl linoleate (grease) were considered to be the unique aroma compounds of the STF.

**TABLE 2:** Odor description of aroma‐active compounds in three samples

No.	Compounds	RIs^a^	Odor description^b^	Previously reported
1	Phenethyl alcohol	1,163	Honey‐like	^2^
2	Furfuryl alcohol	853	Burnt	^2^
3	Benzyl alcohol	1,124	Sweet, flower	^1^
4	Linalool	951	Flower, lavender	^2^
5	1‐Hexanol	836	Floral	^2^
6	2,3‐Butanediol	743	Burning	^2^
7	Alpha‐terpineol	1,220	Oil, anise, mint	^1^
8	1‐Octen‐3‐ol	1,093	Earthy	^9^
9	Phenylacetaldehyde	1,129	Honey‐like	^9^
10	Benzaldehyde	976	Almond, burnt sugar	^3^
11	Furfural	846	Bread, almond, sweet	^4^
12	3(Methylthio)propionaldehyde	943	Sauce, cooked potato	^4^
13	1‐Nonanal	1,158	Floral	^4^
14	Decanal	1,235	Floral, sweet	^4^
15	Isovaleric acid	880	Sweat	^5^
16	Butyric Acid	823	Rancid, cheese, sweat	^2^
17	Ethyl Hexanoate	1,059	Apple peel, fruit	^5^
18	Ethyl laurate	2,134	Leaf	^2^
19	Methyl salicylate	1,211	Peppermint	^2^
20	Ethyl phenylacetate	1,444	Rosy, honey	^7^
21	Ethyl linoleate	2,193	Grease	^7^
22	Ethyl nonanoate	1,519	Coconut	^4^
23	Methyl decanoate	1,504	Grape	^5^
24	4‐Ethylphenol	1,192	Spicy	^6^
25	4‐Ethyl‐2‐methoxyphenol	1,442	Burnt, spicy	^5^
26	2‐Acetyl pyrrole	1,145	Bread, walnut, licorice	^8^
27	2,6‐Dimethylpyrazine	988	Nutty, coffee, green	^8^

Abbreviation: nd, not detected.

^a^ RIs: Calculated in HP‐5 column in GC‐O.

^b^ Odor description at the olfactory detection port.

^c^ Intensity of odorants: 1 (very weak), 2 (weak), 3 (medium), 4 (strong).

^1^ Adedeji et al. (1991).

^2^ Beaulieu and Stein‐Chisholm (2016).

^3^ Triqui and Reineccius (1995).

^4^ Lee and Noble (2003).

^5^ Schnermann and Schieberle (1997).

^6^ Feng et al. (2014).

^7^ Feng et al. (2015).

^8^ Perez‐Boada et al. (2005).

^9^ Morales‐Valle et al. (2011).

^10^ Fanaro et al. (2012).

^11^ Zhao et al. (2011).

^12^ Al‐Dalali et al. (2019).

^13^ Lin et al. (2019).

^14^ Inoue et al. (2016).

As reported in the literatures, the production of fruit aroma is related to ester compounds (Al‐Dalali et al., 2019). Isovaleric acid was found in different foods with a sweaty, strong pungent, and cheesy taste (Zhou et al., 2017). Moreover, the studies had shown that the spiciness was caused by some phenolic compounds such as 4‐ethylphenol and 4‐ethyl‐2‐methoxyphenol in PXDB (Zhang et al., 2020).

### 3.3 OAV analysis of aroma active compounds

As shown in Table 3, the concentrations of 22 volatile compounds exceeded their odor threshold, namely, their OAVs ≥ 1. Among them, the 14 aroma compounds with OAVs > 1 in the three samples were phenyl alcohol, linalool, 1‐hexanol, phenylacetaldehyde, 3‐(methylthio) propionaldehyde, 1‐nonanal, ethyl hexanoate, ethyl laurate, methyl salicylate, ethyl phenylacetate, methyl decanoate, 4‐ethylphenol, and 4‐ethyl‐2‐methoxyphenyl. As reported in the literatures, phenylacetaldehyde known as main volatiles were found to come from free amino acids formed during fermentation (Lin et al., 2019). 3‐methylthiopropanal was a sulfur‐containing compound with a cooked potato, which was also found in other fermented products, such as Korean soy sauce, high‐salt soy sauce, and yeast extraction (Zhao et al., 2011). 4‐ethyl‐2‐methoxyphenol with burnt and spicy aroma was considered as a dominant odor impression of PXDB, which had been reported to be related to the metabolic activity of yeast (Lin et al., 2019). Moreover, esters also played an important role in the aroma profile of PXDB due to their low odor threshold and desirable fruity and sweet odor. The other volatiles with OAVs > 1 are only detected in one or two kinds of samples. For example, five aroma compounds which presented OAVs > 1 only were found in the STF, including 2,3‐butanediol, alpha terpineol, 1‐octen‐3‐ol, benzaldehyde, and ethyl linoleate. But butyric acid with OAV > 1 is only detected in the SGT and STF.

**TABLE 3:** OAV analysis of aroma compounds in three samples

No	Compounds	Odor threshold (µg/kg)	OAV		
			SGT	STF	SCT
1	Phenethyl alcohol	390^1^	2.980	4.011	7.201
2	Furfuryl alcohol	4,500.6^2^	<1	<1	<1
3	Benzyl alcohol	900^3^	<1	<1	<1
4	Linalool	37^4^	3.397	10.154	5.176
5	1‐Hexanol	9^5^	16.644	80.000	20.733
6	2,3‐Butanediol	95.1^2^	<1	6.679	<1
7	alpha‐Terpineol	0.3^6^	nd	744.000	nd
8	1‐Octen‐3‐ol	1^1^	nd	165.400	nd
9	Phenylacetaldehyde	4^1^	114.750	453.850	120.700
10	Benzaldehyde	350^1^	<1	5.656	<1
11	Furfural	3000^1^	<1	<1	<1
12	3‐(Methylthio)propionaldehyde	1.4^2^	64.786	263.857	55.357
13	1‐Nonanal	8^1^	4.575	31.575	8.513
14	Decanal	70.8^7^	nd	<1	nd
15	Isovaleric acid	1200^2^	<1	<1	nd
16	Butyric Acid	3.19^8^	97.586	299.248	nd
17	Ethyl Hexanoate	2.3^2^	72.087	91.304	162.217
18	Ethyl laurate	3.5^8^	75.829	46.000	189.229
19	Methyl salicylate	0.06^8^	1713.333	5,083.333	2,878.333
20	Ethyl phenylacetate	1^8^	117.000	264.900	319.000
21	Ethyl linoleate	4^8^	nd	42.125	nd
22	Ethyl nonanoate	1.2^8^	14.417	nd	35.500
23	Methyl decanoate	1^8^	13.500	47.900	30.700
24	4‐Ethylphenol	140^3^	5.884	12.569	10.017
25	4‐Ethyl‐2‐methoxyphenol	50^1^	21.658	45.992	40.412
26	2‐Acetyl pyrrole	19^1^	18.442	44.089	14.642
27	2,6‐Dimethylpyrazine	10^8^	1.520	nd	<1

Abbreviations: GSTF, gradient steady‐state temperature field; OAV, odor activity value; SCT, samples of constant temperature; STF, samples of traditional fermentation; SGT, samples including samples of GSTF.

^a^ Orthonasal odor thresholds in water determined and taken from the literature:

^1^ Lin et al. (2019).

^2^ Giri et al. (2010).

^3^ Chen et al. (2013).

^4^ Frauendorfer and Schieberle (2006).

^5^ Zhao et al. (2020).

^6^ Gernert (2011).

^7^ Gao et al. (2014).

^8^ Tieman et al. (2006).

Besides, the four aroma compounds with OAVs < 1 in the three samples were furfuryl alcohol, benzyl alcohol, furfural, and decanal. Among them, furfural had the lowest OAV in three samples, which result from its high odor threshold. But the roles of furfural in soy sauce could not be underestimated for furfural could interact with other flavor substances and enhance the taste of soy sauce although furfural had a high threshold (Feng et al., 2014).

### 3.4 Principal component analysis

**FIGURE 7:**
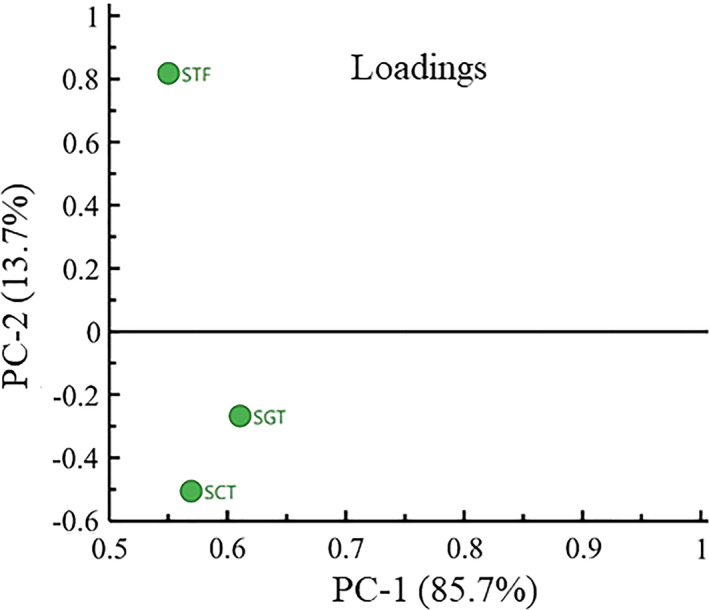
Principal component analysis of major aroma active compounds in three samples

Principal component analysis (PCA) is an unsupervised clustering method that does not require any knowledge of the data set, which reduces the dimensionality of multivariate data while preserving most of the variance therein (Lee & Ahn, 2009). Therefore, the relationships among the 22 major aroma‐active compounds were clarified by PCA as shown in Figure 7. The first principal component (PC‐1) explained 85.7% of the variation across the samples, while the second component (PC‐2) revealed 13.7% of the variation, which represented 99.4% of the total variation. Furthermore, the samples produced by the three fermentation processes had different positions in different loading areas. As shown in Figure 7, the SGT was closer to the STF on the coordinate axis, so the three samples could be divided into two groups in which SCT was divided into a group but SGT and STF were divided into another group indicating that the odor characteristics of the SGT were similar to that of the STF. This PCA results were most partly in accordance with those of aroma‐active compounds analysis and OAV analysis.

### 3.5 Sensory analysis of three kinds of PXDB products

**FIGURE 8:**
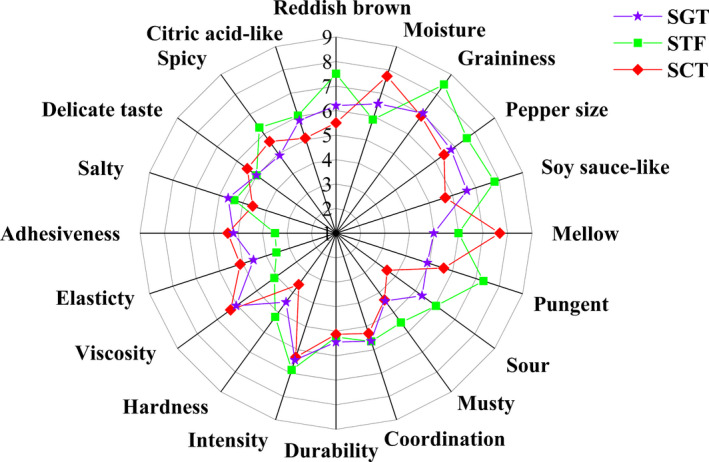
Sensory characteristics of three samples

The sensory quality of the three samples was identified by QDA. The description categories were divided into appearance (reddish brown, moisture, graininess, and pepper size), smell (soy sauce‐like, mellow, pungent, sour, musty, coordination, durability, and intensity), texture (hardness, viscosity, elasticity, and adhesiveness), and taste (salty, delicate taste, spicy, and citric acid‐like). It could be seen from Figure 8, for the appearance, the reddish brown and the moisture score of SGT were closer to those of the STF than those of the SCT, which probably caused by the variable temperature in the GSTF that was closer to that in the traditional fermentation process, resulting in some similarity in color and moisture between the two products. In terms of smell, soy sauce‐like odor scored the highest, indicating that soy sauce‐like odor was the characteristic smell of PXDB. Compared with the SCT, the results showed that the mellow, soy sauce‐like and sour odor of the SGT was closer to the STF, which could be related to some alcohols, aldehydes, and acids, such as 3‐(methylthio)propionaldehyde, isovaleric acid, butyric acid, and phenylethanol. In addition, the scores of coordination, durability, and intensity of the three kinds of PXDB were similar. In terms of texture, the score of the SGT was more similar to the STF, which might be caused by the differences of moisture. In terms of taste, the evaluation results of salty taste, delicate taste, and citric acid‐like taste in the SGT were more similar to those of the STF. In conclusion, compared with the SCT, the sensory characteristics of SGT were more similar to those of the STF, which were consistent with the analyses of OAV and PCA.

**FIGURE 9:**
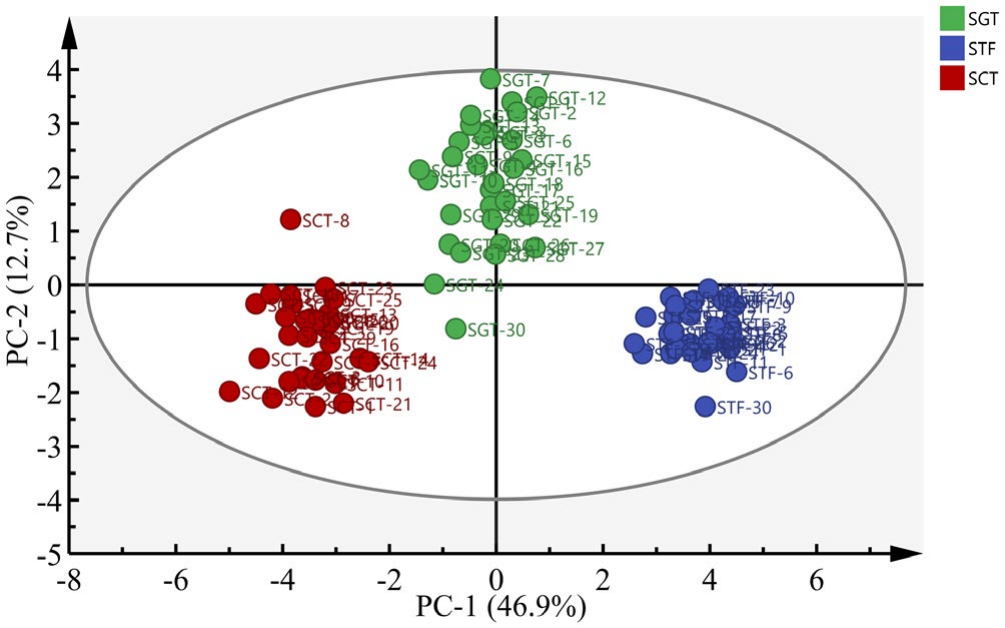
Principal component analysis on sensory evaluation of three samples

The sensory scores for the three samples were analyzed by the PCA as shown in Figure 9. The sensory scores for the three samples given by the 10 evaluators were relatively concentrated, indicating that the consistency and repeatability of the evaluation results were good and the results could truly reflect the sensory characteristics of the three types of products. According to the positions of the three samples, they could be divided into two groups, in which SCT was divided into a group but STF and SGT were divided into another group. The results were consistent with those in Figure 8 and the sensory quality of the SGT was closer to the STF than that of the SCT.

## 4 CONCLUSION

This study focused on the characteristics of the volatile compounds in PXDB produced from different three fermentation processes. A total of 103 volatile compounds were detected in the three samples, of which 75, 67, and 68 volatiles were detected in SGT, SCT, and STF, respectively. Compared with STF, more kinds of esters and alcohols were obtained in SGT by analyzing with GC‐MS although the total concentrations of volatiles in the SGT were smaller, which suggested that the process in the closed system of GSTF was conducive to produce more kinds of esters and alcohols compared with traditional fermentation. A total of 27 active compounds including eight alcohols, six aldehydes, two acids, seven esters, two phenols, and two heterocyclics were detected in the three kinds of samples by GC‐O analysis, of which the three largest major compounds were alcohols, esters, and aldehydes. A total of 22 major aroma‐active compounds were identified in the three samples by the combination analysis with GC‐O and OAV. The PCA results of 22 major aroma‐active compounds had shown that SGT and STF could be divided into a group indicating that the odor characteristics of the SGT were similar to those of the STF. This PCA results were most partly in accordance with those of aroma‐active compounds analysis and OAV analysis. The appearance (reddish brown and moisture), smell (soy‐sauce‐like mellow and sour), texture, and taste (salty, delicate, and citric acid) of sensory index in the SGT exhibited a more similar profile with the STF by the sensory evaluation of QDA and PCA, which had shown that the sensory characteristics of the SGT were more similar to those of the STF. The closed system of GSTF could be applied in PXDB fermentation to obtain higher quality products, which brought a bright prospect of replacing the traditional fermentation process to realize the controllable industrialized production of PXDB.

## CONFLICTS OF INTEREST

The authors declare no conflict of interest.

## ETHICAL APPROVAL

In this research, there are no studies involving animal or human subjects.

## Data Availability

The data that support the findings of this study are not shared.
